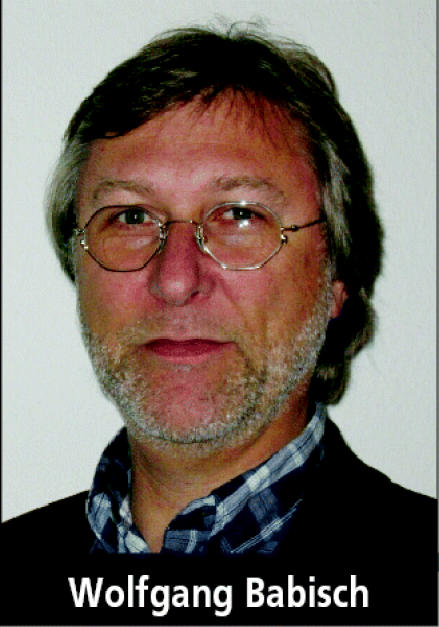# Guest Editorial: Noise and Health

**DOI:** 10.1289/ehp.113-a14

**Published:** 2005-01

**Authors:** Wolfgang Babisch

**Affiliations:** Federal Environmental Agency, Berlin, Germany, E-mail: wolfgang.babisch@uba.de

Noise affects everybody in everyday life—at home, at leisure, during sleep, when traveling, and at work. However, human organisms are not prepared to shut off the noise. Hearing is a permanent process using cortical and subcortical structures to filter and interpret acoustical information; the analysis of acoustical signals is essential for human survival and communication. Noise is detrimental to health in several respects, for example, hearing impairment, sleep disturbance, cardiovascular effects, psychophysiologic effects, psychiatric symptoms, and fetal development (Stansfeld et al. 2000). Furthermore, noise has widespread psychosocial effects including noise annoyance, reduced performance, and increased aggressive behavior [American Academy of Pediatrics 1997; World Health Organization (WHO) 2001].

Noise causes acute mechanical damage to hair cells of the cochlea in the inner ear when the short-term sound intensity or peak impulse noise levels are very high {L_AF_ (A-weighted sound pressure level) > 120 dB; L_Cpk_ (C-weighted peak sound pressure level) > 135 A-weighted decibels [dB(A)]}. In the long run, average sound pressure levels (L_Aeq_) of > 85 dB(A) are likely to cause significant hearing loss due to metabolic exhaustion [International Organization for Standardization (ISO) 1990]. This is not only relevant in occupational settings but also with respect to leisure activities, including firecrackers, toy pistols, and other noisy toys; loud music in discotheques, concerts, and when listening via headphones; and noisy machines and tools (Maassen et al. 2001). Particularly, children and adolescents are affected (Bistrup et al. 2001). The WHO and the U.S. Environmental Protection Agency consider a daily average sound exposure equivalent to L_Aeq_ = 70 dB(A) to be safe for the ear (WHO 2000). The large numbers of young people with hearing impairments should serve as a warning. “Noise hygiene” can be improved, particularly through education at school.

Even ear-safe sound levels can cause nonauditory health effects if they chronically interfere with recreational activities such as sleep and relaxation, if they disturb communication and speech intelligibility, or if they interfere with mental tasks that require a high degree of attention and concentration (Evans and Lepore 1993). The signal–noise ratio (in terms of signal processing) should be at least 10 dB(A) to ensure undisturbed communication. High levels of classroom noise have been shown to affect cognitive performance (Bistrup et al. 2001). Reading and memory have been reported to be impaired in schoolchildren who were exposed to high levels of aircraft noise (Hygge et al. 2002). Some studies have shown higher stress hormone levels and higher mean blood pressure readings in children exposed to high levels of community noise (Babisch 2000; Passchier-Vermeer 2000).

During sleep, electrophysiologic awakening reactions can be detected in an electroencephalogram for event-related maximum noise levels above L_AF_ = 40–45 dB(A) in the bedroom (e.g., aircraft overflights). Recent studies suggest even lower thresholds. The long-term somatic consequences of such arousals are still a matter of discussion and research (WHO Regional Office for Europe 2004). Sleep deprivation, however, is associated with an increased risk of accidents and injuries. Cardiovascular responses found during sleep were independent of sleep disturbance. A subject may sleep during relatively high noise levels but still show autonomic responses.

Among other nonauditory health end points, short-term changes in circulation (including blood pressure, heart rate, cardiac output, and vasoconstriction) as well as in levels of stress hormones (including epinephrine, norepinephrine, and corticosteroids) have been studied in experimental settings for many years (Babisch 2003; Berglund and Lindvall 1995). From this, the hypothesis emerged that persistent noise stress increases the risk of cardiovascular disorders including high blood pressure and ischemic heart disease. Classical biologic risk factors have been shown to be elevated in subjects who were exposed to high levels of traffic noise. Nowadays the biological plausibility of the association is established (Babisch 2002). Its rationale is the general stress concept:

- Sound/noise is a psychosocial stressor that activates the sympathetic and endocrine systems.
- Acute noise effects do not occur only at high sound levels in occupational settings, but also at relatively low environmental sound levels when, more importantly, certain activities such as concentration, relaxation, or sleep are disturbed.

The following questions need to be answered:

- Do these changes observed in the laboratory habituate, or do they persist under chronic noise exposure?
- If they habituate, what are the physiologic costs; if they persist, what are the long-term health effects?

There is no longer any need to prove the noise hypothesis as such. Decision making and risk management rely on quantitative risk assessment, but not all biologically notifiable effects are of clinical relevance. The results of epidemiologic noise studies suggest an increase in cardiovascular risk with increasing noise exposure (e.g., Babisch 2000). Unfortunately, most of the individual studies that have been carried out lack statistical power. Over the years the quality of studies has improved, and many potential confounding factors have been considered. Some expert groups have rated the evidence of an association as sufficient (overview by Babisch 2002; Passchier-Vermeer 2003). Transportation noise from road and air traffic is the predominant sound source in our communities; outdoor sound levels for day–evening–night (L_den_) > 65–70 dB(A) were found to be associated with odds ratios of 1.2–1.8 in exposed subjects compared with unexposed subjects [< 55–60 dB(A)] (Babisch 2000). Because large parts of the population are exposed to such noise levels [European Environmental Agency (EEA) 2004], noise policy can have a significant impact on public health (Kempen et al. 2002; Neus and Boikat 2000). For noise levels below an L_den_ of 55 dB(A), no major annoyance reactions or adverse health effects are to be expected.

Studies use magnitude of effect, dose–response relationship, biological plausibility, and consistency of findings among studies as issues in epidemiologic reasoning. Environmental and health policy must determine acceptable noise standards that consider the whole spectrum from subjective well-being to somatic health. This means that limit values may vary depending on the severity of outcomes. Future noise research should focus on source-specific differences in risk characterization, combined effects, differences between objective (sound level) and subjective (annoyance) exposure on health, sensitive/vulnerable groups, sensitive periods of the day, coping styles, and other effect-modifying factors.

## Figures and Tables

**Figure:**